# Effects of Wearing a 50% Lower Jaw Advancement Splint on Biophysical and Perceptual Responses at Low to Severe Running Intensities

**DOI:** 10.3390/life12020253

**Published:** 2022-02-08

**Authors:** Filipa Cardoso, Ana S. Monteiro, João Paulo Vilas-Boas, João Carlos Pinho, David B. Pyne, Ricardo J. Fernandes

**Affiliations:** 1 Centre of Research, Education, Innovation and Intervention in Sport, CIFI2D, Faculty of Sport, University of Porto, 4200-450 Porto, Portugal; up201406202@edu.fade.up.pt (A.S.M.); jpvb@fade.up.pt (J.P.V.-B.); ricfer@fade.up.pt (R.J.F.); 2 Porto Biomechanics Laboratory, LABIOMEP-UP, Faculty of Sport, University of Porto, 4200-450 Porto, Portugal; 3 Faculty of Dental Medicine, University of Porto, 4200-393 Porto, Portugal; jpinho@fmd.up.pt; 4 Institute of Science and Innovation in Mechanical and Industrial Engineering, INEGI, Faculty of Engineering, University of Porto, 4200-465 Porto, Portugal; 5 Research Institute for Sport & Exercise, University of Canberra, Canberra 2617, Australia; david.pyne@canberra.edu.au

**Keywords:** occlusal splints, jaw advancement, outdoor running, oxygen uptake, ventilation, running kinematics

## Abstract

Acute ergogenic effects of wearing occlusal splints have been reported for aerobic and anaerobic exercises, but the literature centered on performance improvement by using jaw repositioning splints is scarce. We aimed to analyze the effect of wearing a 50% lower jaw advancement splint on biophysical and perceptual responses at low to severe running intensities. Sixteen middle- and long-distance runners performed twice a 7 × 800 m intermittent running protocol (with 1 km·h^−1^ increments and 30 s rest periods) in an outdoor track field using two lower intraoral splints (a placebo and a lower jaw advancer). These devices were custom manufactured for each participant and a randomized and repeated measure design was used to compare conditions. No differences between placebo and lower jaw advancer were found (e.g., 52.1 ± 9.9 vs. 53.9 ± 10.7 mL·kg^−1^·min^−1^ of oxygen uptake, 3.30 ± 0.44 vs. 3.29 ± 0.43 m of stride length and 16 ± 3 vs. 16 ± 2 Borg scores), but small effects were sometimes observed (e.g., 109.2 ± 22.5 vs. 112.7 ± 25.2 L·min^−1^ of ventilation, ES = −0.42). Therefore, this jaw advancement splint had no substantial ergogenic effect on biophysical and perceptual responses when running at different intensities.

## 1. Introduction

Ranging from sprint to ultramarathon, running performance depends on the time required to cover a given distance and results from the interplay between physiological and biomechanical determinants [1,2,3]. Since middle- and long-distance performances are mainly dependent on runners’ maximal oxygen uptake, exercise economy and technical skills [1,2,3], some preliminary studies aimed to understand how the dental occlusion and the mandibular position may impact running exercise. In this regard, occlusal splints have been manufactured particularly aiming for muscular strength, endurance and lower limb kinematic improvements [4,5,6] and generic use for overall performance enhancement.

Mouthguards have been developed over the years to protect the teeth and the orofacial structures against contact-sports-related injuries. However, recent evidence on both aerobic [5,7,8] and anaerobic [9,10,11] performance increments have led to a renewed interest in dental splints for sport applications. Although several fitted splints have been presented recently, changing the mandibular position could yield positive effects on respiratory gas exchange, breathing mechanism and running pattern [4,5,7]. These biophysical changes might be related to the fact that the mandibular forwarding position leads to a wider cross-sectional upper airway area and to adjustments in breath, swallowing and lips closure [5,12,13]. It is unclear how far to advance the lower jaw to achieve ergogenic exercise effects, and more satisfactory outcomes seem to be obtained by dragging the jaw to a more prominent forward position (established at 50% of the maximal protrusion range position) [14].

Despite the above-referred performance benefits of wearing occlusal splints during sport, studies have reported conflicting findings, particularly regarding the use of jaw repositioning devices. Since further research is required to clarify the likely outcomes on improving exercise performance, we have aimed to analyze the effect of wearing a 50% lower jaw advancement splint on the biophysics of running across a wide range of intensities. In addition, we aimed to assess the runners’ perceptual responses regarding exercise effort, breathability and comfortability when performing from low to severe intensities using the advancement splint. We have hypothesized that wearing a 50% jaw advancement splint would enhance running gas exchange, improve lower limb linear kinematics, reduce the sense of effort and make it easier to breathe within the established exercise intensity spectrum. 

## 2. Materials and Methods

### 2.1. Participants

Sixteen middle- and long-distance male runners volunteered to participate in the current study. Their main anthropometric and training background characteristics were: age 29.6 ± 7.4 years, body mass 70.5 ± 8.9 kg, height 179.3 ± 7.0 cm, middle- and long-distance running practice 12.1 ± 5.3 years and weekly running training of 12.0 ± 3.6 h. Participants were recruited via personal contact or their sporting coach and based on the following eligibility criteria: (i) without a history of cardiac, respiratory, metabolic, physical diseases or injuries within the previous three months; (ii) without severe dental or periodontal disease; (iii) without current orthodontic treatment and (iv) having ≥ two years of running training background. All the experiments were approved by the local Ethics Committee (code: CEFADE282020) and participants (or parent/guardian for the participants under 18 years old) read and signed an informed consent form in accordance with the Declaration of Helsinki. 

### 2.2. Dental Splints Manufacturing

Two lower intraoral splints (a placebo and a lower jaw advancer) were custom manufactured according to the oral anathomical characteristics of each participant after obtaining upper and lower dental impressions (alginate, orthoprint, Zhermach, Badia Polesine, Italy) and stone models (gypsumm, Crystacal R, Saint-Gobain Formula, Newark, UK). Both splints were laboratory-made by a dentist using a vacuum thermoformer machine (EcoVac, Clarben, Madrid, Spain) and clear thermoforming foils (Clarben, Madrid, Spain). Placebo splints did not modify the mandibular position and were trimmed on the occlusal surfaces to ensure no interference on each runner’s dental occlusion and occlusal vertical dimension [14]. 

To develop the mandibular repositioning splint, the absolute range of maximal mandibular protrusion was determined for each participant using a George Gauge apparatus (Great Lakes Orthodontics, Ltd., New York, NY, USA) and a 5 mm interincisal vertical opening bite fork. Then, a silicone bite registration (Occlufast Rock, Zhermack, Badia Polesine, Italy) was taken with the lower jaw placed at 50% of the maximal protrusion range position [14,15]. Posteriorly, the dental stones were mounted on a semi-adjustable articulator (A7 Plus, Bio-Art, São Paulo, Brasil) and acrylic bite plates were attached to the thermofoiling foils to produce dental edentations, allowing subjects to keep an advanced mandibular position. 

### 2.3. Experimental Protocol

**Figure 1:**
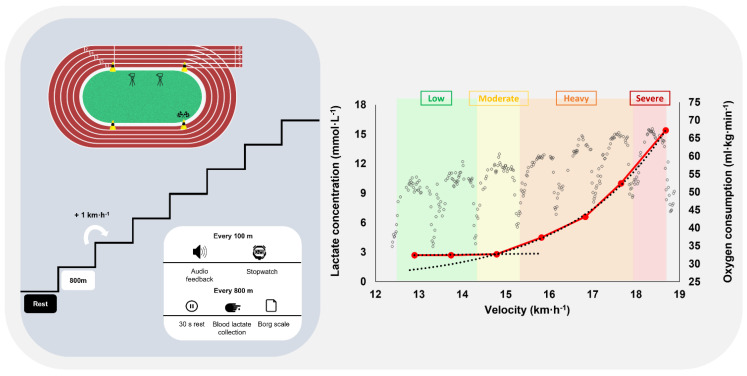
Scheme of the implemented outdoor incremental intermittent running protocol (left panel) and example of the blood lactate concentrations and oxygen consumption kinetics along the running protocol allowing to establish the low to severe intensity domains (right panel).

Runners were familiarized with all testing procedures, equipment and splints prior to each experimental session. They were asked to wear the same running shoes during experiments and to bite the intraoral splint when performing the trial with the 50% advancement device. After an individualized running warm-up (of ~20 min duration at low intensity), participants performed an incremental intermittent running protocol of seven stages of 800 m (with 1 km·h^−1^ increments and 30 s rest periods in-between) on a 400 m outdoor track field (Figure 1, left panel) [16]. The participants’ initial velocity was set according to their own experience in previous tests or to their best individual 800 m performance at that time. Runners were also instructed to control their velocity in each stage according to audible feedbacks emitted when they crossed fluorescent cones placed on the track field at every 100 m [16]. The protocol was performed twice (48 h apart), single-blind and randomly assigned for the two different splint conditions. 

During the experimental protocol, breath-by-breath gas exchange variables were measured continuously by a telemetric (previously calibrated) portable gas analyzer (K4b^2^; Cosmed, Rome, Italy). It was comfortably attached to the runners back, placed near to their body’s center of mass to avoid relevant interferences with their running technique. Split times at each 100 m were obtained using a stopwatch (Seiko, Tokyo, Japan) and capillary blood samples (5 μL, Lactate Pro2; Arkay, Inc., Kyoto, Japan) for blood lactate analysis were collected from the fingertip at baseline, during the 30 s rest periods and at the 1st, 3rd, 5th and 7th min of the recovery period [16,17]. Lower limb kinematics were also recorded at 120 Hz by two high-definition video cameras (GoPro HERO6 Black, San Mateo, CA, USA) calibrated before each testing session and placed between the 200–300 m section of the running track (Figure 1, left panel).

The rate of perceived exertion assessment was collected at the end of each stage through direct feedback from the runners and using the 6–20-point Borg scale [18]. To obtain the participants’ perceptions toward the splint’s use, a set of questions (“I feel that the splint limited my breath”, “I feel wearing the splint limited my performance”, “I feel the splint uncomfortable” and “I would consider the use of the splint during my sports practice”) was adapted from the modified Athletic Mouthguard Attitude Questionnaire and the literature [19,20] and completed at the end of the running protocol using a sample 5-point Likert scale. 

### 2.4. Data Analysis

The collected ventilatory breath-breath data were edited to exclude errant breaths from coughing or signal interruptions. Only data between mean ± 3 standard deviations were included for analysis, being posteriorly smoothed using a moving and time average of three breaths and 10 s, respectively [21]. The mean values from the last 30 s of exercise were used for comparison between experimental conditions, with conventional physiological criteria being applied to define the maximal oxygen uptake [22]. The lactate–velocity curve modelling method, through the determination of interception point of the best fit of a combined linear and exponential pair of regressions (Figure 1, right panel), was used to determine each runner’s anaerobic threshold [14,23]. Using the maximal oxygen uptake and the anaerobic threshold as physiological indicators, the low, moderate and heavy (the stages under, at and above the anaerobic threshold, respectively) and severe (the stage where maximal oxygen uptake was elicited) exercise intensity domains were established (Figure 1, right panel) [21,24].

Running velocity was assessed by dividing the 800 m distance by the times accomplished at each running stage and was similar between placebo vs. 50% lower jaw advancement splint experimental conditions at the low, moderate, heavy and severe running intensity domains: 13.5 ± 2.9 vs. 13.4 ± 2.7, 15.3 ± 2.9 vs. 15.4 ± 2.7, 17.1 ± 2.7 vs. 17.2 ± 2.7 and 18.1 ± 2.5 vs. 18.1 ± 2.6 km·h^−1^, respectively. Kinematic assessment was performed based on the images from the specified 100 m section of the track field using a valid, reliable and free motion analysis software (Kinovea, v. 0.8.27, Boston, MA, USA) [14,25]. A total of six strides were analyzed to obtain the stride time (the time between the left-foot touchdown and the next left-foot touchdown), frequency (1/stride time) and length (running velocity/stride frequency) [26,27]. The mean values of perceived exertion and the mean scores of each participant’s perceptions toward the splint-use questions were determined for each exercise intensity domain.

### 2.5. Statistical Analysis

Statistical procedures were completed using SPSS (version 27.0.1.0, IBM Corp., Armonk, NY, USA), with normal data distribution checked for all variables using the Shapiro–Wilk test. Standard statistical methods were employed for calculating the mean and standard deviation for all the assessed biophysical and perceptual variables. Pairwise comparisons between splints were conducted for each exercise intensity domain (*p* ≤ 0.05 level) and Cohen’s d effect size (ES) was computed to compare the magnitude of changes between experimental conditions (trivial <0.2, small 0.2–0.6, moderate 0.6–1.2, large 1.2–2.0 and very large >2.0) [28]. A sample size of 19 participants was required for a paired sample design (one-tailed test) to detect a medium effect with a 5% significance level and 80% power using G*power statistical software.

## 3. Results

### 3.1. Physiological Measurements

**Figure 2:**
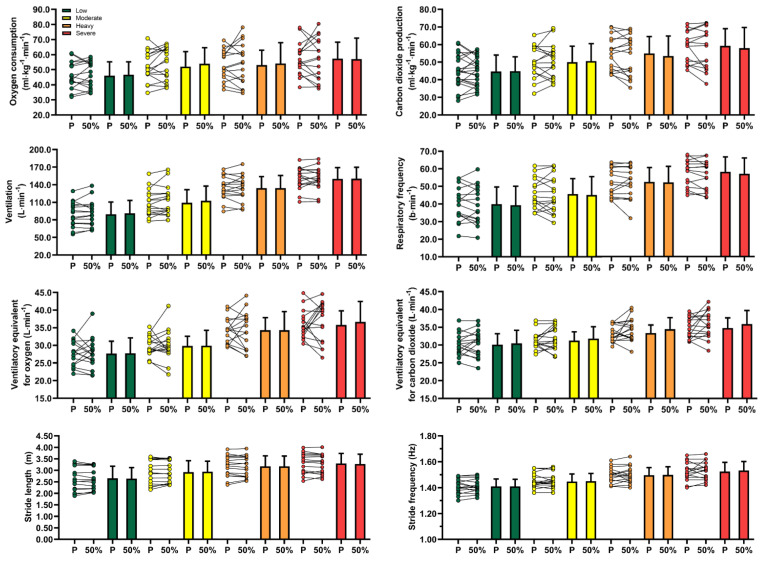
Individual (coloured circles), mean (coloured bars) and standard deviation (error bars) values of the biophysical variables assessed during the incremental protocol for the placebo (P) and 50% lower jaw advancement (50%) splints across low (green), moderate (yellow), heavy (orange) and severe (red) exercise intensity domains.

The selected physiological variables did not differ between experimental conditions across the low to severe running intensity domains (Figure 2). Several small effects were identified when comparing placebo and 50% advancement splints, particularly oxygen consumption at moderate intensity (−0.30), carbon dioxide production at heavy and severe intensities (0.30 and 0.27), ventilation at low and moderate intensities (−0.24 and −0.42), respiratory frequency at severe intensity (0.41) and ventilatory equivalent for carbon dioxide at moderate, heavy and severe intensities (−0.24, −0.33 and −0.34).

### 3.2. Kinematic Measurements

Kinematic variable values also did not differ when running with placebo and advancement splints across the studied intensity domains. Similar to the physiological measurements, small ES values were found when comparing both experimental conditions but only for stride length at moderate and severe running intensities (−0.25 and 0.27). Trivial effects were observed for stride length at low and heavy intensity domains (0.13 and 0.02) and for stride rate throughout the entire intensity spectrum (−0.03, −0.11, −0.06 and −0.19 for low, moderate, heavy and severe domains, respectively). 

### 3.3. Perceptual Responses 

**Figure 3:**
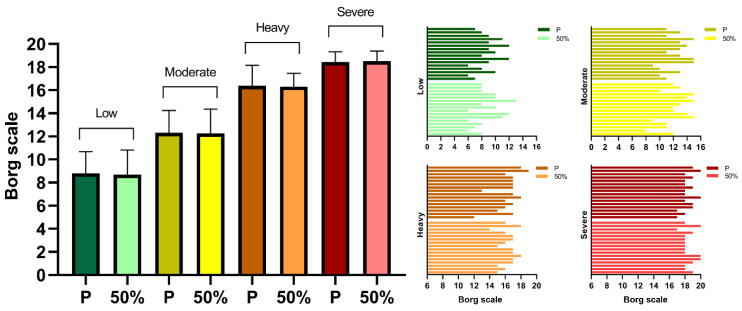
Borg scale scores mean, standard deviation and individual values (left and right panels, respectively) assessed during the running low (dark and light green), moderate (dark and light yellow), heavy (dark and light orange) and severe (dark and light red) intensity domains for placebo (P) and 50% lower jaw advancement (50%) splints.

**Figure 4:**
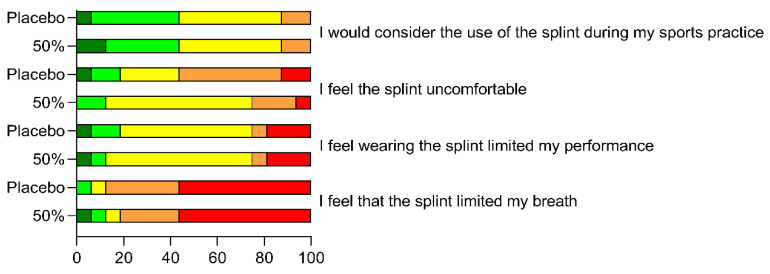
Rating towards the splint’s breathability, comfortability and exercise performance for each question assessed, presented as percentage regarding the 5-point Likert scale (1—strongly agree in dark green, 2—agree in light green, 3—neutral in yellow, 4—disagree in orange and 5—strongly disagree in red).

The perceptual responses concerning exercise effort, as well as breathability, comfortability and exercise performance, when running with both splints, are displayed in Figure 3 and Figure 4. No Borg scale differences were observed between placebo and mandibular advancement conditions across the four running intensities and only trivial ES were displayed (0.09, 0.06, 0.04 and 0.06 for low, moderate, heavy and severe domains, respectively). An overall positive perception was reported for breathability when wearing placebo and 50% advancement splints, with 88% and 81% of the runners admitting not feeling any breath impairments during the incremental protocol. Few subjects considered that the splints impaired their performance, with most of them being neutral (56% and 63% for placebo and 50% advancement devices) or in disagreement/strong disagreement (25% for both conditions) regarding the negative influence on running. Similarly, a small percentage of participants were uncomfortable using our splints and 44% considered the possibility of wearing them in their sport practice. 

## 4. Discussion

The main purpose of the current study was to analyze the biophysical changes of wearing a 50% advancement splint while running from low to severe intensities. Concurrently, it was aimed to observe runners’ perception regarding exercise effort and the splint’s breathability and comfortability. Compared with a placebo splint, our laboratory-made 50% lower jaw advancement device did not affect the studied running physiological and kinematic variables. Furthermore, the runners’ perception of effort, breathability and comfortability across the above-referred wide spectrum of exercise intensities were similar between splint conditions.

The improvement of airway volumes by wearing mandibular advancement devices (frequently ranging from 50% to 75% of mandible protrusion) is well established in clinical settings and used as a first-line therapy for mild to moderate obstructive sleep apnea to prevent upper airway collapse during sleep [29,30,31]. Based on the assumptions and positive outcomes from the sleep apnea patients’ treatment, mandibular advancement splints (with modest design changes) have also been manufactured for sport purposes [5,13,14]. Since there are ergogenic effects on gas exchange when using a mandibular advancement splint, which seem to be closely related to airway improvements, it can be assumed that its use would be more beneficial for subjects with narrow airways and acute/chronic respiratory diseases. Concurrently, its effects have been confirmed in healthy and trained subjects [5,13,14].

Without compromising oxygen uptake during exercise, hyperventilation and lower respiratory rates have been collectively associated with lower breathing work and substantially lower airway resistance to airflow [5,32,33]. However, under the influence of different intraoral splints, conflicting and variable findings were described, such as lower ventilation values [7,13], higher respiratory frequency [14] and increased/decreased oxygen uptake values [7,14]. The current study did not identify any positive contribution from the used advanced splint for gas-exchange variables, not confirming the higher ventilatory responses observed when using other lower jaw forwarding splints [5,14]. These divergent findings are likely related to variations regarding intraoral splint construction, sample characteristics, experimental protocols and exercise modes/intensities.

In the current study, there is no specific information on the effect of the used advancement oral device on airway structural changes, it not being clear if there was a relationship between the degree of mandibular movement and the upper-airway dimensions. A case study with mandibular splints at different protruding ranges (30% and 50%) evidenced a positive biophysical effect on running performance-related variables [14] but, since airway dimensional changes appear to depend on craniofacial development [30], higher inter-individual deviations might be observed at a larger sample size (due to airway structural differences between subjects). The absence of physiological improvements when using the 50% lower jaw advancement splint in the current study might be partly explained by these subject-specific variations.

In addition, and even if we standardized the teeth incisal thickness with the 5 mm George Gauge bite fork, the real incisal opening given by the 50% advancement splint was not the same for all runners, since it depends on their maximum intercuspation position (varying if they have a normal, deep or open bite). We have observed that some runners needed to increase their open mouth (particularly along the heavy and severe running intensities) more than the amplitude given by the intraoral advancement device, resulting in a non-bite on the splint. This could be a plausible justification for the lack of substantial differences between the placebo and 50% advancement devices, especially at running intensities above the anaerobic threshold where there is an evident transition between mainly aerobic and a mix aerobic/anaerobic energy supplies [23,34].

Several mandibular amplitude oscillations are produced during our daily activities and exercise routines. The mandibular position remains quite stable during sitting or standing, but small amplitude oscillations occur during walking and large forces and quick movements are applied to the mandible during running [35,36,37]. Wearing intraoral splints during running exercise could possibly induce individual specific changes in the movement pattern [4], but this remains controversial and unclear. Although previous findings reported linear kinematic improvements during a treadmill running incremental protocol by wearing 30% and 50% protruding splints [14], and a more symmetrical movement pattern during running on a level walkway under the influence of different mandibular splints [4], the eventual positive influence of changing lower jaw position in running kinematics was not confirmed by us. Nevertheless, this is the first study to test the effect of wearing a mandibular protruding splint during a real running context.

Understanding each runner’s perception was of great value to evaluate the splint’s use at track field running conditions, with our 50% advancement splint not affecting scores through the different intensity domains. Despite the fact that the 50% advancement device is bulkier than the placebo, the runners’ perception of the ability to breath was similar between intraoral devices. A large percentage of subjects did not report that wearing the splints impaired their performance, although only ~40% admitted considering its use during their sport routines. Given the similar perceptual responses towards exercise effort when using the splints, we believe that the 50% advancement device did not have a negative influence, not seriously interfering with running exercise. Some of the negative perceptions regarding wearing the intraoral devices may be related to the absence of sufficient familiarization with them [38].

We acknowledge there are some limitations with this investigation. Clearly it is important to assess the facial skeletal pattern in future research to better control possible large variations between subjects. Complementarily, as the influence of changing mandibular position may differ from subject to subject, individual athlete assessment should also be considered to understand the influence of mandibular position change during exercise. Furthermore, since our study was slightly underpowered, we also consider it important to confirm our findings in studies employing a larger sample size, involving both female and male subjects. In upcoming studies, wearing the splints for a few days during the participants actual sport training should be considered for long-term familiarization. Finally, as the range of motion while running is much higher than during walking, and kinematic changes induced by intraoral splints can be difficult to detect, a sophisticated motion capture system and data analysis need to be applied in further research.

## 5. Conclusions

Our laboratory-made 50% advancement splint had no substantial ergogenic effect on physiological, kinematic and perceptual responses while running at a track field at different intensities. However, runners reported no impairments on their exercise performance when the advancement splint was worn. The lack of detrimental effects and likelihood of performance benefits of wearing mandibular repositioning splints could encourage the sport practitioners to further test its utility in training settings. Further research is required in a large cohort of sport practitioners, both in field and laboratory conditions and in different exercise modes to confirm the effects of jaw advancement splints in sports.

## Data Availability

The dataset generated and analyzed during the current study is available from the corresponding author on reasonable request.
